# Predictive value of tumor mutational burden for PD-1/PD-L1 inhibitors in NSCLC: A meta-analysis

**DOI:** 10.1097/MD.0000000000034990

**Published:** 2023-10-06

**Authors:** Wenjie Li, Yanjun Zhao, Hongjun Zhang, Wenying Zheng, Ruixuan Wang, Xing Gu

**Affiliations:** a Department of Respiratory and Critical Care Medicine, Xi’an Chest Hospital, Chang’an District, Xi’an, Shanxi, China.

**Keywords:** PD-1/PD-L1 inhibitors, biomarker, meta-analysis, non-small cell lung cancer, tumor mutational burden

## Abstract

**Background:**

To investigate the association between tumor mutational burden (TMB) and the therapeutic effect of Programmed Death 1/Programmed Death Ligand 1 inhibitors in non-small cell lung cancer.

**Methods:**

Four electronic databases, PubMed, Embase, Web of Science, and Cochrane Library, were searched on May 10, 2023, and no time limitation was applied. Analyses were performed using STATA17.0. We assessed the methodological quality of each randomized controlled trial using the Newcastle-Ottawa scale.

**Results:**

After exhaustive database search and rigorous screening, 10 studies were included in the meta-analysis. Our findings indicate that high TMB significantly improves progression-free survival but reduces overall response rate. The overall survival was not significantly different between the high and low TMB groups. No significant publication bias was observed.

**Conclusion:**

High TMB serves as a potential predictive biomarker for improved progression-free survival and reduced overall response rate in patients with non-small cell lung cancer treated with programmed death 1/programmed death ligand 1 inhibitors. However, its predictive value in overall survival requires further investigation.

## 1. Introduction

Lung cancer is a leading cause of cancer-related mortality worldwide and continues to pose a significant global health challenge. Non-small cell lung cancer (NSCLC) accounts for a substantial proportion of lung cancer cases, contributing to approximately 9.3% of global cancer incidence and 14.6% of cancer-related deaths.^[1]^ Despite advancements in the field, the prognosis for NSCLC remains poor, with a meager 5-year survival rate of approximately 17.4%. Traditional treatment modalities, including surgery, chemotherapy, radiation therapy, and biological therapy, often fail to yield satisfactory outcomes, underscoring the critical need for novel therapeutic approaches.^[2,3]^

The advent of immunotherapy has revolutionized the therapeutic landscape of many cancers, including NSCLC. Specifically, the programmed death 1/programmed death ligand 1 (PD-1/PD-L1) pathway has become a promising target, offering hope for improved outcomes.^[4]^ However, not all patients exhibit favorable responses to PD-1/PD-L1 inhibitors, highlighting the importance of identifying predictive biomarkers to facilitate individualized treatment strategies. One such biomarker that has garnered considerable attention is tumor mutational burden (TMB), a quantitative measure of the total number of coding errors, base substitutions, and insertions or deletions in the protein-coding regions of a tumor genome. TMB, an indicator of neoantigen load and genomic instability, is positively correlated with immunotherapy outcome. A high TMB potentially signifies a higher number of neoantigens, which may boost the immune system’s ability to recognize and attack tumor cells, thereby enhancing the efficacy of immunotherapies such as PD-1/PD-L1 inhibitors.^[4,5]^

However, the precise role and utility of TMB as a predictive biomarker for the efficacy of PD-1/PD-L1 inhibitors in NSCLC remain a matter of ongoing debate.^[6]^ While several studies have suggested a positive correlation between high TMB and improved clinical response to PD-1/PD-L1 blockade, others have reported conflicting findings, highlighting the need for further investigation of the complex interplay between TMB, immune response, and therapeutic efficacy in NSCLC.^[7,8]^

Therefore, this study aimed to conduct a systematic review and meta-analysis to compare the clinical efficacy of PD-1/PD-L1 inhibitors in patients with NSCLC with varying TMB levels. By elucidating the role of TMB in influencing the response to PD-1/PD-L1 inhibitors, we hope to provide a more robust foundation for their utilization in the treatment of advanced NSCLC, potentially leading to more personalized treatment strategies and improved clinical outcomes.

## 2. Materials and Methods

During the systematic review process and subsequent reporting of our results, we adhered to the Preferred Reporting Items for Systematic Reviews and Meta-Analyses guidelines.^[9]^ As the information utilized in this article was sourced from published materials, there was no need for informed consent or ethical approval. Two researchers conducted a systematic search for pertinent studies, independently determined their eligibility, extracted data, and evaluated the quality of the research. The 2 researchers were required to reach consensus and resolve any points of disagreement.

### 2.1. Search strategy

Four electronic databases, PubMed, Embase, Web of Science, and Cochrane Library, were searched on May 10, 2023, and no time limitation was applied. The vocabulary and syntax were adapted according to the database. PubMed search terms were as follows: (Non-small Cell Lung Cancer “Non-small Cell Lung Cancer”[MeSH Terms] odds ratios (OR) NSCLC[Title/Abstract] OR “non-small cell lung cancer”[Title/Abstract] OR “non-small cell lung carcinoma”[Title/Abstract] OR “lung adenocarcinoma”[Title/Abstract] OR “lung squamous cell carcinoma”[Title/Abstract]) AND (“Programmed Cell Death 1 Receptor” [MeSH Terms] OR PD-1 [Title/Abstract] OR “Programmed Death 1”[Title/Abstract] OR “Programmed Cell Death Protein 1”[Title/Abstract] OR “PD1”[Title/Abstract]) AND (“Programmed Cell Death 1 Ligand 1 Protein”[MeSH Terms] OR PD-L1[Title/Abstract] OR “Programmed Death Ligand 1”[Title/Abstract] OR “PDL1”[Title/Abstract]) AND (“Programmed Cell Death 1 Receptor/antagonists & inhibitors”[MeSH Terms] OR “PD-1 inhibitors”[Title/Abstract] OR “PD-L1 inhibitors”[Title/Abstract]) AND (“Tumor Mutational Burden”[MeSH Terms] OR TMB [Title/Abstract] OR “Tumor Mutational Burden”[Title/Abstract] OR “Mutational Load”[Title/Abstract] OR “Genomic Instability”[Programmed]). No language limitations were applied.

### 2.2. Inclusion criteria

Studies included in the systematic review were required to meet the following criteria: Studies involving patients with pathologically confirmed NSCLC; Clinical trials or cohort studies that utilized TMB with a defined cutoff value to assess outcomes in NSCLC patients treated with PD-1/PD-L1 inhibitors (nivolumab, pembrolizumab, atezolizumab, durvalumab, and avelumab); Studies that provided OR for objective response rate/total response rate (ORR), hazard ratios (HR) for progression-free survival (PFS) or overall survival (OS), and their corresponding 95% confidence intervals (CIs), or those that provided sufficient information to extract these data; and studies with an evaluable patient population of at least 20 individuals.

The exclusion criteria were as follows: Repeatedly published literature; Documents with incomplete or unclear analytical data and inconsistent outcome indicators; Documents with poor quality and lack of original data; and; Studies in which it was impossible to extract data on the relationship between TMB and outcome indicators in NSCLC patients treated with PD-1/PD-L1 inhibitors.

### 2.3. Data extraction

The literature screening and data extraction shall be carried out independently by 2 evaluators and cross-checked; if there are differences, the differences will be discussed and resolved. The data to be extracted included author(s), year of publication, phase of the trial, line of treatment, investigational drugs, number of patients with high TMB and low TMB, PFS and its 95% confidence interval, ORR and its 95% CI, OS and 95% CI. When there was no data of interest in the published report, we contacted the investigators of the original study by email to request unpublished data.

### 2.4. Quality assessment

Two independent reviewers assessed the quality of the included studies using the Newcastle-Ottawa scale,^[10]^ which comprises 9 components distributed across 3 categories. These categories evaluate the potential sources of bias, including selection, comparability, and outcome. Each study was assigned a quality score ranging from 0 to 9. Studies scoring between 0 to 3 were categorized as low-quality, those with a score of 4 to 6 were considered medium-quality, and those achieving a score of 7 to 9 were classified as high-quality. This structured quality assessment approach ensured a robust and consistent evaluation of the included studies.

### 2.5. Statistical analyses

Heterogeneity between studies was assessed using chi-square statistics and qualified according to the size of *I*^2^. An *I*^2^ value of 0% implied no observed heterogeneity and values > 50% indicated substantial heterogeneity. The publication bias of meta-analyses with ≥ 10 eligible papers was examined using the symmetry of the funnel plot and Egger test. If the funnel plot was asymmetrical, hypothetical negative unpublished studies were imputed to determine whether publication bias significantly affected impact estimates. A 2-sided *P* < .05 was considered statistically significant in all statistical tests. Stata version 17 (StataCorp, College Station, TX) was used for the data analysis.

## 3. Results

### 3.1. Search results and study selection

**Figure 1.**
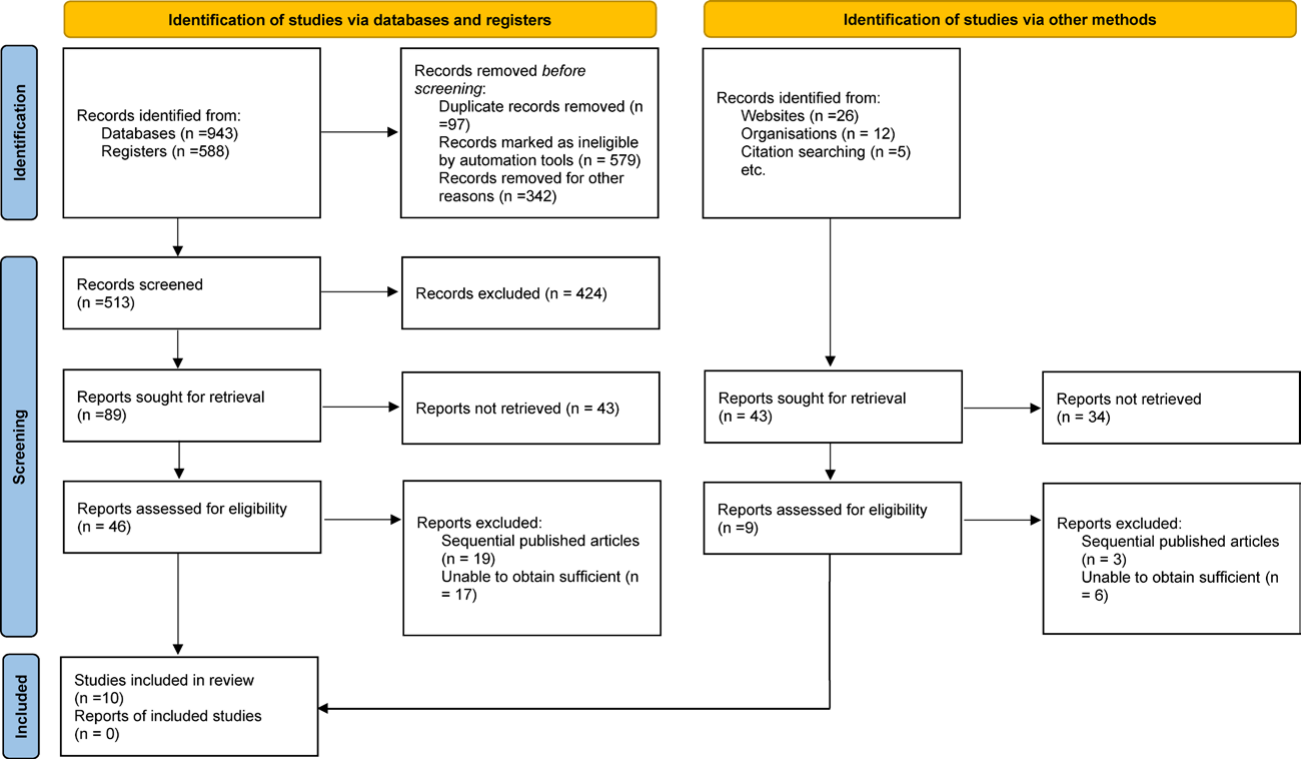
Selection process of included studies.

From the initial search of the electronic databases, 1531 related literatures were found. After removing repetitive literature, reading titles and abstracts, and screening strictly according to the inclusion and exclusion criteria, 28 related studies were obtained and 18 were excluded from further reading. Finally, 10 articles were included.^[6,8,11–18]^ The literature screening process and the results are shown in Figure 1.

### 3.2. Study characteristics

This meta-analysis incorporated a variety of studies examining the efficacy of different experimental drugs, predominantly in America and Asia, with a single European study. These drugs primarily focus on PD-1/PD-L1 inhibitors and their combinations. The TMB cutoff values vary significantly across studies, with some not specifying a cutoff value. These studies primarily used Targeted Next-Generation Sequencing as the detection method, with a few using whole exome sequencing (WES). The sample size evaluated for TMB also varied widely across the studies. The key outcomes measured were PFS, OS, and ORR (Table 1).

**Table 1:** Characteristics of studies included in the meta-analysis.

Authors	Area	Experimental Drugs	TMB Cutoff Value	Detection method	Sample size evaluable for TMB	Outcomes
Rizvi 2015	America	Pembrolizumab	178	WES	34	PFS
Rizvi 2018	America	Mono or combo	The 50th percentile of TMB	Targeted NGS	240	PFS
Hellmann 2018	America	Nivolumab plus ipilimumab	158 mutations	WES	75	PFS, ORR
Chae 2018	America	Anti-PD-1/PD-L1 therapies	15	Targeted NGS	34	PFS, OS
Wang 2019	Asian	Anti-PD-1/PD-L1 therapies	6	Targeted NGS	50	PFS, ORR
Ready 2019	America	Nivolumab plus low-dose ipilimumab	10	Targeted NGS	98	PFS, ORR
Fang 2019	Asian	Anti-PD-(L)1 monotherapy	NA	Targeted NGS	75	PFS, ORR
Chae 2019	America	Anti-PD-1/PD-L1 therapies	NA	Targeted NGS	20	PFS, OS
Alborelli 2020	Europe	Nivolumab/Pembrolizumab/ Atezolizumab/Nivolumab + Ipilimumab	9mut/Mb	Targeted NGS	76	PFS, OS
Huang 2020	Asian	PD-1/PD-L1 inhibitor monotherapy	10mut/Mb	Targeted NGS	34	PFS, OS, ORR

Combo = antiPD-(L)1+anti-cytotoxic T-cell lymphocyte-4 combination therapy, Mono = anti-programmed death 1 or anti-programmed death ligand 1 [anti-PD-(L)1] monotheism, mut = mutation, NA = not available, NGS = next-generation sequencing, ORR = objective response rate, OS = overall survival, PD-1/PD-L1 = programmed death 1/programmed death ligand 1, PFS = progression-free survival, TMB = tumor mutation burden, WES = whole exome sequencing.

### 3.3. Quality assessment

We assessed the methodological quality of each randomized controlled trial using the Newcastle-Ottawa scale. In general, 1 study scored 7 points, 2 studies scored 8 points, and 7 studies scored 9 points. No studies were blinded and there was no evidence of allocation concealment. No funding bias was evident in any of the studies. No studies had incomplete outcome data, early stoppage bias, or baseline imbalances. The risks of bias and corresponding ratios are summarized (Table 2).

**Table 2:** The quality assessment according to NOS of each cohort study.

Study	Selection				Comparability	Outcome			Total score
	Representativ-eness of the exposed cohort	Selection of the non -exposed cohort	Ascertainment of exposure	Demonstration that outcome	Comparability of cohorts	Assessment of outcome	Was follow-up long enough	Adequacy of follow-up of cohorts	
Rizvi 2015	★	★	★	★	★★	★	★	★	9
Huang 2020	★	★	★	★	★★	★	★	★	9
Wang 2019	★	★		★	★	★	★	★	7
Hellmann 2018	★	★	★	★	★★	★		★	8
Alborelli 2020	★	★	★	★	★★	★	★	★	9
Fang 2019	★	★	★	★	★	★	★	★	8
Ready 2019	★	★	★	★	★★	★	★	★	9
Rizvi 2018	★	★	★	★	★★	★	★	★	9
Chae 2018	★	★	★	★	★★	★	★	★	9
Chae 2019	★	★	★	★	★★	★	★	★	9

NOS = Newcastle-Ottawa Scale.

### 3.4. The effectiveness of PD-1/PD-L1 inhibitors based on tumor mutational burden levels

**Figure 2.**
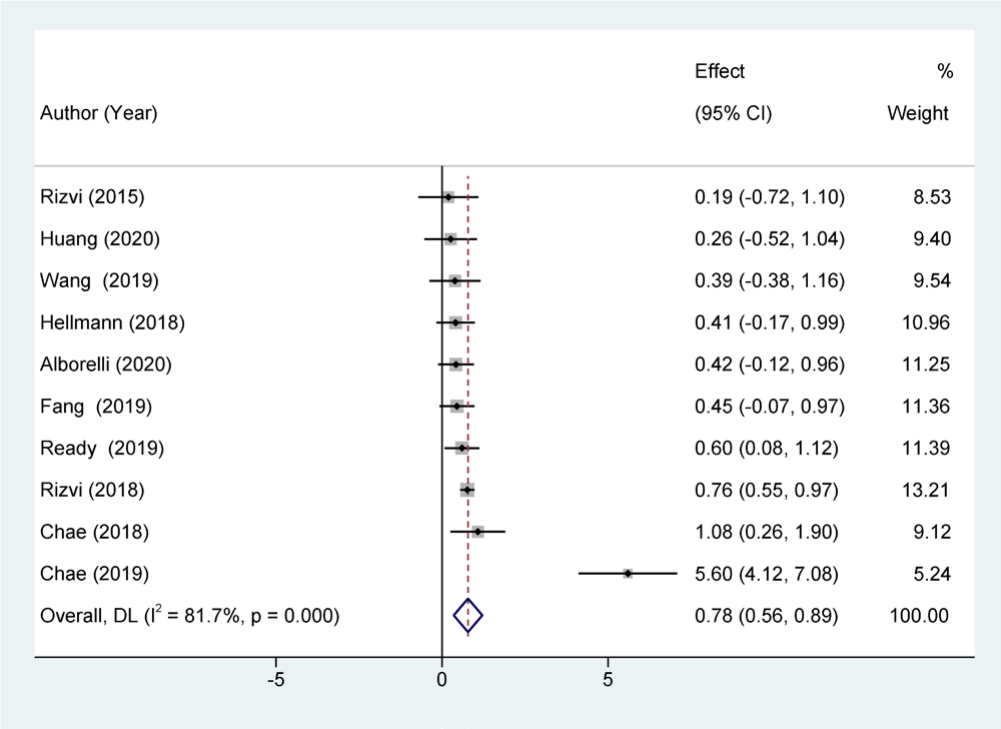
Forest plot of association between TMB and PFS. PFS = progression-free survival, TMB = tumor mutational burden.

**Figure 3.**
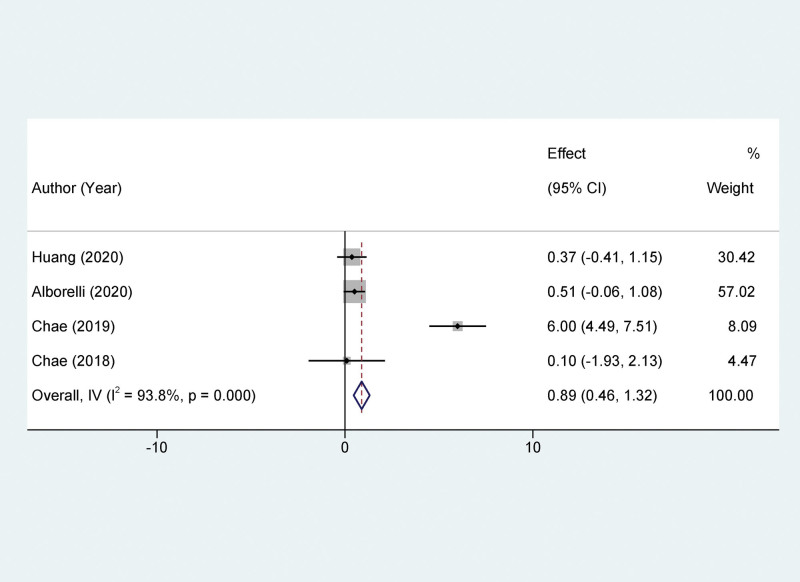
Forest plot of association between TMB and OS. OS = overall survival, TMB = tumor mutational burden.

**Figure 4.**
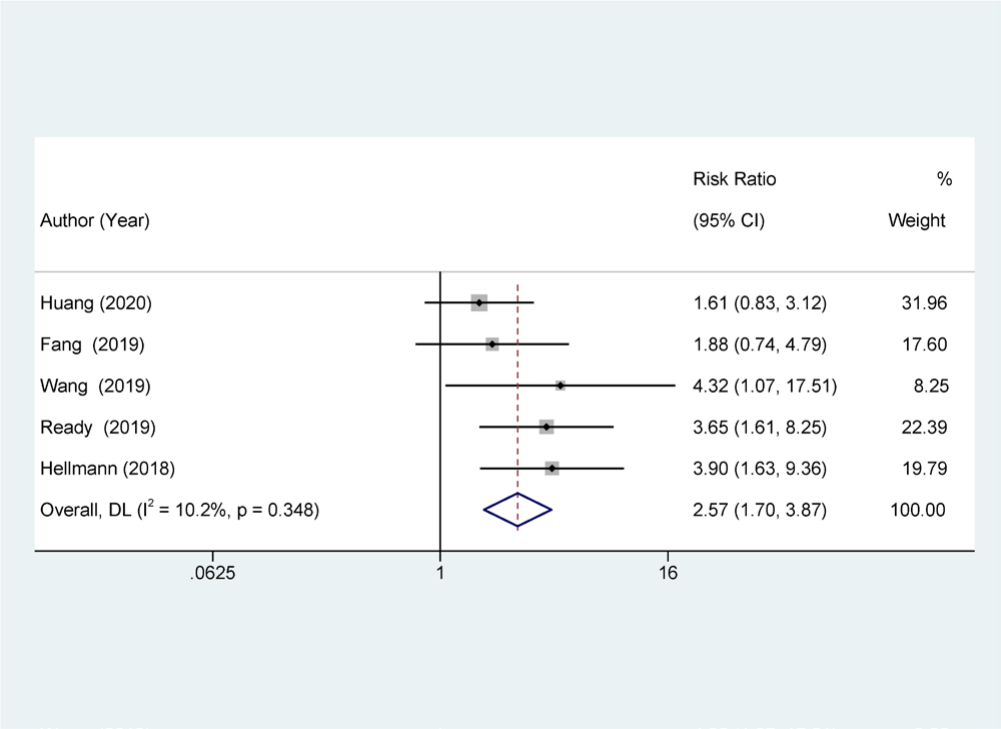
Forest plot of association between TMB and ORR. ORR = overall response rate, TMB = tumor mutational burden.

Our study indicates that compared to patients with low TMB, those with high TMB show significantly improved PFS after treatment with PD-1/PD-L1 inhibitors, yet exhibit a lower ORR. Ten studies reported a relationship between TMB and PFS. The high TMB group had a significantly better PFS than the low TMB group, with a statistically significant difference (HR = 0.0.78, 95% CI: 0.56–0.89; *P* < .001), as depicted in Figure 2. Four studies assessed the relationship between TMB and OS in NSCLC patients treated with PD-1/PD-L1 inhibitors. The difference in OS between the high and low TMB groups was not statistically significant (HR = 0.89, 95% CI: 0.46–1.32, *P* = .26), as shown in Figure 3. Five studies evaluated the correlation between TMB and ORR in NSCLC patients treated with PD-1/PD-L1 inhibitors. The ORR in patients with high TMB was significantly lower than in patients with low TMB, with a statistically significant difference (HR = 2.57, 95% CI: 1.70–3.87; *P* < .001), as depicted in Figure 4.

### 3.5. Publication bias

**Figure 5.**
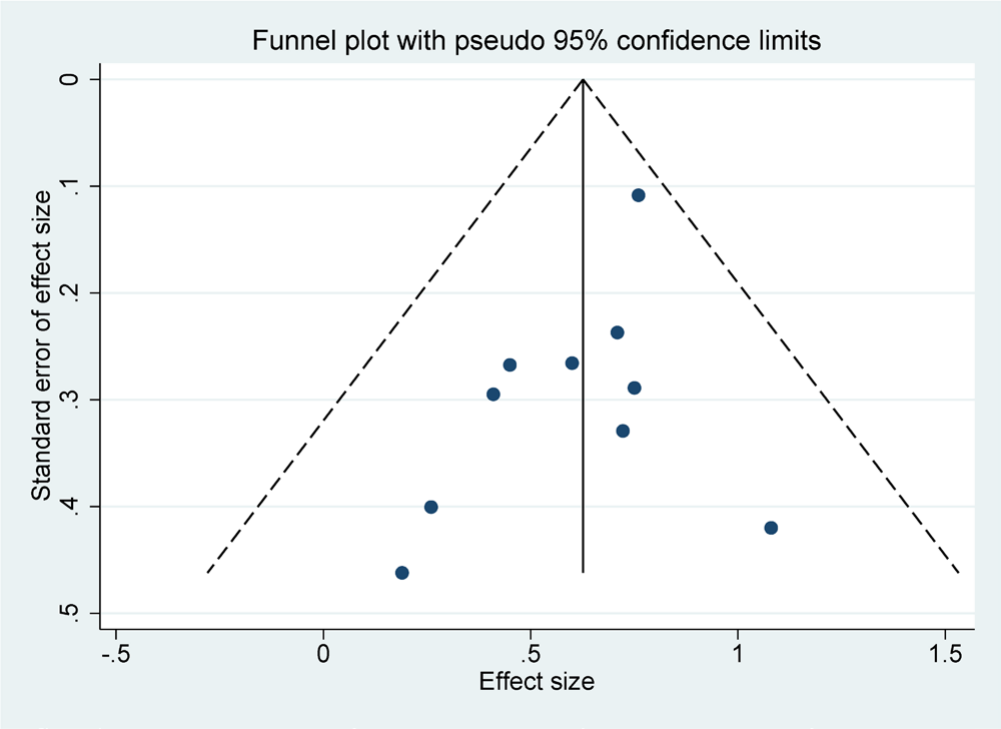
Funnel plot for publication bias in all included studies.

The funnel plots constructed in the observed study showed symmetry, and no significant publication bias was detected in the funnel plots (Fig. 5).

## 4. Discussion

The predictive value of TMB as a biomarker for the clinical efficacy of PD-1/PD-L1 inhibitors in the treatment of NSCLC has attracted increasing scientific attention. This systematic review and meta-analysis delved deeper into this topic, exploring the complex interplay between TMB and immune response modulation in the context of NSCLC. Research has indicated that in the absence of PD-1/PD-L1 inhibitors, patients with high TMB have a worse survival rate, highlighting the clinical value of these inhibitors in improving survival and overcoming poor prognostic characteristics.^[15]^ Furthermore, several studies have indicated that TMB levels are among the highest in NSCLC among various cancers, and TMB is emerging as a potential biomarker for predicting the efficacy of PD-1/PD-L1 inhibitors.^[19]^

Our results demonstrated a positive correlation between high TMB and PFS in immunotherapy, but a negative correlation with ORR. We postulate that this scenario may suggest that TMB is indeed related to the effectiveness of immunotherapy, and that the treatment is very effective in some patients, but not all patients with high TMB can benefit. Although our study identified a correlation between high TMB and lower ORR, this does not necessarily mean that high TMB directly results in reduced ORR. We suggest that this might reflect complex biological mechanisms and immune interactions. For instance, tumors with high TMB may produce more neoantigens, potentially eliciting a stronger immune response but also possibly activating more immune suppression mechanisms, thereby affecting the efficacy of PD-1/PD-L1 inhibitors. Furthermore, high TMB might reflect greater genomic instability, which could influence tumor response to immunotherapy. Such potential complexity indicates the need for deeper investigation into the relationship between TMB and the effectiveness of PD-1/PD-L1 inhibitors to fully understand their interplay. Moreover, increasing clarity is being provided in research on the association of eligible biomarkers, such as PD-1/PD-L1 expression,^[20]^ tumor-infiltrating lymphocytes,^[21]^ oncogenic driver mutations,^[22]^ mismatch repair deficiencies,^[23]^ and microsatellite instability,^[24]^ with the efficacy of PD-1/PD-L1 inhibitors in treating NSCLC. If TMB could be combined with these biomarkers, it might enable a more precise identification of NSCLC patients who could genuinely benefit from PD-1/PD-L1 inhibitors.

TMB refers to the total number of substitutions and insertions/deletions in the exons encoding regions of the assessed genes in the tumor cell genome.^[25]^ On the 1 hand, driver gene mutations can lead to the occurrence of tumors. However, a large number of cell mutations can generate new antigens that can activate CD8 + cytotoxic T-cells, thereby exerting T cell-mediated antitumor effects.^[26]^ Therefore, as the number of gene mutations increases, more new antigens are produced, increasing the likelihood of recognition by the immune system. Activation of the PD-1/PD-L1 pathway can inhibit T lymphocyte proliferation and immune function in T cells.^[27]^ Hence, many scholars believe that TMB may predict the clinical efficacy of PD-1/PD-L1 inhibitors and have conducted extensive research in this direction.

Goodman et al^[28]^reported a strategy for dividing TMB into 3 tiers: low (1–5 mut/Mb), medium (6–19 mut/Mb), and high (≥20 mut/Mb). In clinical practice, it is important to distinguish between high and low TMB expression. The majority of studies had approximately 10 mut/Mb or 150 mutations, which appears to have a relatively stable predictive value in NSCLC. In fact, the variation in TMB under different detection methods is considerable, and there may not be a universal TMB cutoff value suitable for all detection methods.^[29]^ WES cannot currently be used as a predictive factor for treatment response to PD-1/PD-L1 inhibitors, mainly because of its complexity, high cost, and time-consuming nature, which limits its usefulness in everyday clinical practice.^[30]^ Targeted Next-Generation Sequencing is more convenient and cost-effective than WES. Research has shown that the accuracy of TMB determined by targeted NGS decreases in panels with genomic coverage of 0.5.^[25]^ Both targeted NGS and WES require a large number of tumor tissue samples, which is not only invasive but also problematic when the patient’s tumor is too small to obtain a specimen. A time-saving and convenient method for blood-based TMB detection has been reported.^[31]^ Furthermore, clinical trials assessing TMB in solid tumors are underway, and these trials are expected to provide more high-quality results, assisting in determining appropriate TMB cutoff values and detection modes.

This study has some limitations. First, the sample size included in the study varied, resulting in significant discrepancies in sample sizes across different subgroups. Studies with smaller sample sizes could potentially contribute to publication bias in the meta-analysis. Additionally, some crucial clinical characteristics, such as lifestyle, age, and sex, which have been reported as important factors affecting the therapeutic effect of PD-1/PD-L1 inhibitors, have been overlooked owing to insufficient data. While some studies have reported that TMB can independently predict the efficacy of PD-1/PD-L1 inhibitors, our results suggest that this viewpoint requires further research. In our meta-analysis, most of the recruited patients were from the West, where studies showed a strong correlation between high TMB and improved immunotherapy outcomes. Further parallel research is needed in Asia and other regions.

In conclusion, high TMB can predict an increase in PFS after PD-1/PD-L1 inhibitor treatment for NSCLC, but its predictive value for OS, ORR, and long-term survival requires further research. More large-scale and standardized studies are needed to further explore the predictive value of TMB in specific subgroups. Second, there is an urgent need to identify the optimal cutoff value and detection method. Moreover, combining TMB with eligible biomarkers may expand the selection of patients who may benefit from immune checkpoint inhibitors.

## 5. Conclusion

High TMB serves as a potential predictive biomarker for improved PFS and reduced ORR in patients with NSCLC treated with PD-1/PD-L1 inhibitors. However, its predictive value in OS requires further investigation. Future studies should focus on determining the optimal TMB cutoff value, detection methods, and combining TMB with other biomarkers to improve patient selection for immunotherapy.

## Acknowledgements

We appreciate the technical support provided by the hospital.

## Author contributions

**Data curation:** Yanjun Zhao.

**Formal analysis:** Hongjun Zhang.

**Investigation:** Wenying Zheng.

**Software:** Ruixuan Wang.

**Writing – original draft:** Wenjie Li.

**Writing – review & editing:** Xing Gu.
